# Supplementary material for: Association of Household Income Level with Vitamin and Mineral Intake
Source: Nutrients. 2021 Dec 23;14(1):38. doi: 10.3390/nu14010038 (PMC8746700; doi:10.3390/nu14010038)
Supplement: Supplementary file 1 [file nutrients-14-00038-s001.zip › nutrients-1492119-supplementary.pdf]

Table S1. Association between household income quintile and nutrient intake suitability.

Variables	Household income quintile												*p*-Value
	Total		Quintile 1 (very low)		Quintile 2 (low)		Quintile 3 (medium)		Quintile 4 (high)		Quintile 5 (very high)		
	N	%	N	%	N	%	N	%	N	%	N	%	
**Total (n=5088)**	**5088**	**100**	**765**	**15.0**	**946**	**18.6**	**1001**	**19.7**	**1159**	**22.8**	**1217**	**23.9**	
Overall Vitamin uptake suitability													0.004
No	4647	91.3	714	93.3	879	92.9	917	91.6	1054	90.9	1083	89.0	
Yes	441	8.7	51	6.7	67	7.1	84	8.4	105	9.1	134	11.0	
Overall Mineral uptake suitability													<0.001
No	3878	76.2	643	84.1	740	78.2	750	74.9	871	75.2	874	71.8	
Yes	1210	23.8	122	15.9	206	21.8	251	25.1	288	24.8	343	28.2	
Vitamin A uptake suitability													<0.001
No	3864	75.9	629	82.2	750	79.3	759	75.8	859	74.1	867	71.2	
Yes	1224	24.1	136	17.8	196	20.7	242	24.2	300	25.9	350	28.8	
Vitamin B1 uptake suitability													0.004
No	1612	31.7	280	36.6	307	32.5	321	32.1	358	30.9	346	28.4	
Yes	3476	68.3	485	63.4	639	67.5	680	67.9	801	69.1	871	71.6	
Vitamin B2 uptake suitability													<0.001
No	1688	33.2	390	51.0	371	39.2	293	29.3	335	28.9	299	24.6	
Yes	3400	66.8	375	49.0	575	60.8	708	70.7	824	71.1	918	75.4	
Vitamin B3 uptake suitability													<0.001
No	2653	52.1	502	65.6	525	55.5	519	51.8	571	49.3	536	44.0	
Yes	2435	47.9	263	34.4	421	44.5	482	48.2	588	50.7	681	56.0	
Vitamin B9 uptake suitability													<0.001
No	2909	57.2	508	66.4	569	60.1	569	56.8	648	55.9	615	50.5	
Yes	2179	42.8	257	33.6	377	39.9	432	43.2	602	51.9	602	49.5	
Vitamin C uptake suitability													<0.001
No	3617	71.1	597	78.0	708	74.8	707	70.6	815	70.3	790	64.9	
Yes	1471	28.9	168	22.0	238	25.2	294	29.4	344	29.7	427	35.1	
Ca uptake suitability													<0.001

No	3649	71.7	631	82.5	708	74.8	689	68.8	809	69.8	812	66.7	
Yes	1439	28.3	134	17.5	238	25.2	312	31.2	350	30.2	405	33.3	
P uptake suitability													<0.001
No	847	16.6	242	31.6	175	18.5	153	15.3	155	13.4	122	10.0	
Yes	4241	83.4	523	68.4	771	81.5	848	84.7	1,004	86.6	1,095	90.0	
Fe uptake suitability													0.001
No	1601	31.5	240	31.4	279	29.5	344	34.4	399	34.4	339	27.9	
Yes	3487	68.5	525	68.6	667	70.5	657	65.6	760	65.6	878	72.1	

The number and percentage of participants from each group, and the p-values are shown.

BMI: Body Mass Index.

[a] Defined as the sum of the number of chronic diseases from: hypertension, dyslipidemia, stroke, myocardial infarction, angina pectoris, and diabetes mellitus.

[b] BMI values were used to determine obesity status. $0 < BMI < 18.5$; underweight, $18.5 \leq BMI < 23.0$; normal, $23.0 \leq BMI < 25.0$; overweight, $BMI \geq 25.0$; obese.
